# ConiferEST: an integrated bioinformatics system for data reprocessing and mining of conifer expressed sequence tags (ESTs)

**DOI:** 10.1186/1471-2164-8-134

**Published:** 2007-05-29

**Authors:** Chun Liang, Gang Wang, Lin Liu, Guoli Ji, Lin Fang, Yuansheng Liu, Kikia Carter, Jason S Webb, Jeffrey FD Dean

**Affiliations:** 1 Department of Botany, Miami University, Oxford, Ohio 45056, USA; 2 Department of Automation, Xiamen University, Xiamen, Fujian, 361005, China; 3 Beijing Genomics Institute, Beijing 101300, China; 4 Warnell School of Forestry and Natural Resources, University of Georgia, Athens, Georgia 30602, USA

## Abstract

**Background:**

With the advent of low-cost, high-throughput sequencing, the amount of public domain Expressed Sequence Tag (EST) sequence data available for both model and non-model organism is growing exponentially. While these data are widely used for characterizing various genomes, they also present a serious challenge for data quality control and validation due to their inherent deficiencies, particularly for species without genome sequences.

**Description:**

ConiferEST is an integrated system for data reprocessing, visualization and mining of conifer ESTs. In its current release, Build 1.0, it houses 172,229 loblolly pine EST sequence reads, which were obtained from reprocessing raw DNA sequencer traces using our software – WebTraceMiner. The trace files were downloaded from NCBI Trace Archive. ConiferEST provides biologists unique, easy-to-use data visualization and mining tools for a variety of putative sequence features including cloning vector segments, adapter sequences, restriction endonuclease recognition sites, polyA and polyT runs, and their corresponding Phred quality values. Based on these putative features, verified sequence features such as 3' and/or 5' termini of cDNA inserts in either sense or non-sense strand have been identified *in-silico*. Interestingly, only 30.03% of the designated 3' ESTs were found to have an authenticated 5' terminus in the non-sense strand (*i.e*., polyT tails), while fewer than 5.34% of the designated 5' ESTs had a verified 5' terminus in the sense strand. Such previously ignored features provide valuable insight for data quality control and validation of error-prone ESTs, as well as the ability to identify novel functional motifs embedded in large EST datasets. We found that "double-termini adapters" were effective indicators of potential EST chimeras. For all sequences with *in-silico *verified termini/terminus, we used InterProScan to assign protein domain signatures, results of which are available for in-depth exploration using our biologist-friendly web interfaces.

**Conclusion:**

ConiferEST represents a unique and complementary public resource for EST data integration and mining in conifers by reprocessing raw DNA traces, identifying putative sequence features and determining and annotating *in-silico *verified features. Seamlessly integrated with other public resources, ConiferEST provides biologists powerful tools to verify data, visualize abnormalities, including EST chimeras, and explore large EST datasets.

## Background

Although a relatively small taxonomic group in terms of species numbers, conifers are the dominant plants in many terrestrial ecosystems. Better knowledge of their genomic structure and function, thus, carries immense possibilities for improving our understanding of the ecological drivers of their evolution, as well as our genetic approaches to sustainable commercial forestry. Conifer genomes are usually large and replete with highly repetitive sequences. For example, loblolly pine (*Pinus taeda*), a North American conifer that provides approximately 16% of the worlds' annual timber supply, has a haploid genome size of about 22 pg (*e.g*., about 7× larger than the human genome), of which at least 50–60% could be characterized as highly repetitive DNA. Clearly, such a genome would present a serious challenge to complete genome sequencing using present technologies. As an alternative, ESTs continue to be a dominant approach for characterizing the active, protein-coding portions of conifer genomes [1-3].

**Figure 1:**
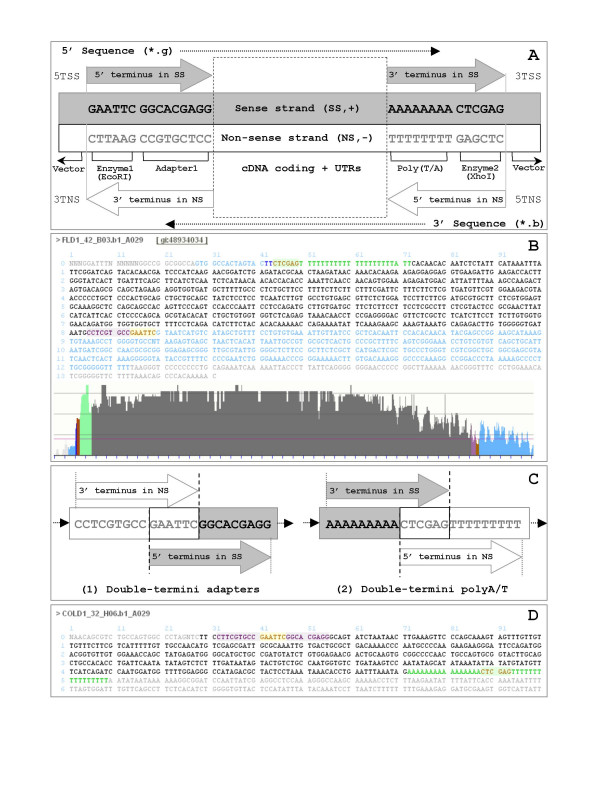
**Terminology adopted by ConiferEST with examples**. **Panel A**: Illustration of the expected structures of cDNA library constructs as interpreted in ConiferEST. 5' terminus in SS (5TSS): → GAATTC(EcoRI) + GGCACGAGG(Adapter1) →. 3' terminus in SS (3TSS): → PolyA + CTCGAG(XhoI) →. 5' terminus in NS (5TNS): → CTCGAG(XhoI) + PolyT →. 3' terminus in NS (3TNS): → CCTCGTGCC(Adapter1) + GAATTC(EcoRI) →. SS stands for sense strand and NS for non-sense strand. **Panel B**: An example of a 3'-end EST sequence that fits the expected structures. FLD1_42_B03.b1_A029 is a 3'-end sequence showing a complete cDNA insert flanked by vector sequence at each end. The zoomable bar graph represents the Phred quality profile for each nucleotide base. The dark black text/bar indicates base calls within a good quality region determined using moving windows and a threshold of Phred quality value 16, whereas light black text/bar indicates calls of lower quality. PolyA/T tails are shown in green. Dark blue is used to highlight vector sequence in a good quality region, while light blue indicates vector sequence of lower quality. Sequence consistent with Adapter1 is highlighted in pink. Restriction endonuclease recognition sites for EcoRI and XhoI are brown with yellow background and brown with green background, respectively. **Panel C**: Two types of "double-termini assembly" (*i*.*e*., "double-termini adapters" and "double-termini polyA/T") that might result in chimeric sequences. **Panel D**: A supposed 3'-end sequence (COLD1_32_H06.b1_A029) is actually a 5'-end sequence, with "double-termini adapters" and "double-termini polyA/T" at each end.

**Figure 2:**
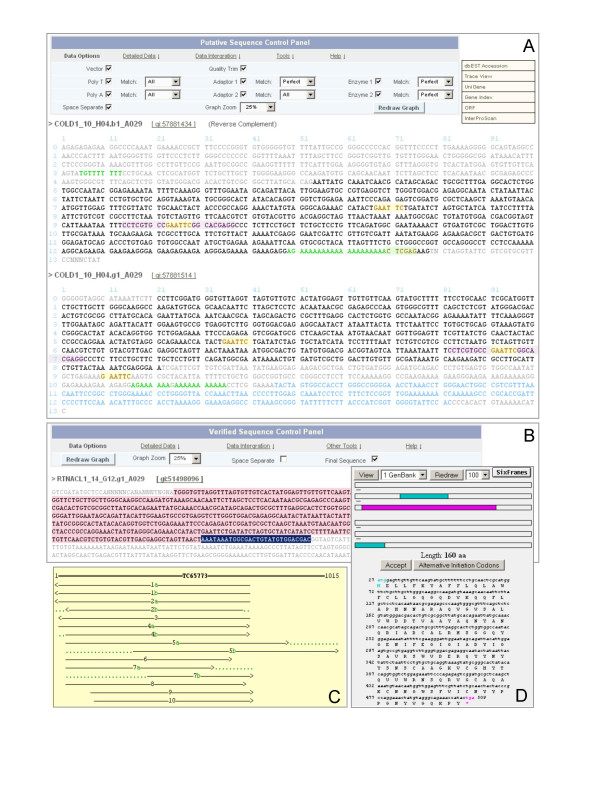
**Using web interfaces to identify chimeric EST sequences and clusters**. **Panel A**. When the user clicks the *Reverse Complement *menu item displayed when the *Detailed Data *link in the *Putative Sequence Control Panel *is selected, a reverse complement view will be shown. COLD1_10_H04.b1_A029 is a 3'-end sequence with a verified 5' terminus in NS (or 3' terminus in SS in reverse complementary view) and "double-termini adapters". Its 5' counterpart, COLD1_10_H04.g1_A029, also has "double-termini adapters" and a detectable polyA tail. **Panel B**. Like *Putative Sequence Control Panel*, *Verified Sequence Control Panel *provides users many options for customizing their sequence views but focuses on verified features. For a given sequence, checking the *Final Sequence *box within the *Verified Sequence Control Panel *and then clicking *Redraw Graph *button, highlights with a red background the final sequence, which can then be directed to other search tools or cut-and-pasted into other applications. RTNACL1_14_G12.g1_A029 is a 5'-end sequence without any verified terminus. The last 28 bases (*i*.*e*., AAATAAATGGCGACTGTATGTGGACGAC, the bases with black background) of its final sequence have been manually highlighted with the cursor for illustration purpose. **Panel C**. Clicking the *Gene Index* menu item displayed when the *Data Integration *link in *Putative/Verified Sequence Control Panel *is selected, pops-up the relevant Gene Index cluster view. As shown, all three above-mentioned sequences are found in cluster TC65773, where COLD1_10_H04.g1_A029 is labelled as "***5a***", COLD1_10_H04.b1_A029 as "***5b***", and RTNACL_14_G12.g1_A029 as "***8***" within the cluster alignment graph. To verify the alignment, we found the last 28 bases of RTNACL1_14_G12.g1_A029 were located from 867 to 894 in COLD1_10_H04.b1_A029 (reverse complement) and from 635 to 662 in COLD1_10_H04.g1_A029. It appears that the whole cluster obtains about 300 extra bases in its 3' end because of the double-termini adapters. **Panel D**. By clicking the *ORF *menu item available after the *Data Integration *link in *Putative/Verified Sequence Control Panel *is selected, the final sequence for a given sequence read will be dynamically sent out for open reading frame detection. As shown, RTNACL1_14_G12.g1_A029 displays 6-frame ORF results. If available, the user can follow the *InterproScan *menu item that appears when the *Data Integration *link is selected to explore relevant protein signatures of the InterPro member databases.

ESTs are primarily single-pass cDNA sequences derived from mRNAs. They provide a snapshot of gene expression for a defined biological sample at a given developmental stage as a function of prevailing environmental conditions. ESTs are used for a wide variety of genome characterization approaches, including gene discovery and gene expression studies [4,5], detecting putative polymorphisms [6], building genetic/physical maps [7], and annotation of genomic sequence [8]. Indeed, the success of these applications depends heavily on the quality of EST sequences, which cannot be guaranteed due to artifacts of cDNA library construction (*e.g*., inversely inserted and chimeric cDNAs) and inherent errors in DNA sequencing procedures [9,10]. Variously modified oligo-dT primers are commonly used to prime reverse transcription of the mRNAs to create inserts for cDNA libraries. Correspondingly, as illustrated in Figure 1A, a typical approach to construct a directionally cloned cDNA library leaves an adapter/linker sequence immediately adjacent to specific recognition site(s) for restriction endonuclease. In theory, as a consequence of 3'-end sequencing, 3' sequence reads should contain at least a 5' terminus in the non-sense strand (*i.e*., 5TNS containing a polyT tail), as well as a 3' terminus in the non-sense strand (*i.e*., 3TNS). Similarly, if the cDNA insert is short enough the 5'-end sequence reads should contain at least a 5' terminus in the sense-strand (*i.e*., 5TSS) and occasionally a 3' terminus in the sense-strand (*i.e*., 3TSS containing a polyA tail) (see Figure 1A, 1B). In practice, however, inserts do not always match these expected structures. In some cases, imperfections in molecular biology manipulations may result in such artifacts as multiple and/or concatenated adapters/linkers within sequence reads (see Figures 1C, 1D, 2A). Alternatively, DNA sequencing issues, such as sequencing primers positioned too close to the ends of cDNA inserts or variable base-calling quality in single-pass sequencing, can yield sequences with unexpected structures.

Many public web-based sequence resources (*e.g*., NCBI GenBank and EMBL Nucleotide Sequence Database) have been developed to address the quality, redundancy and less-than-full-length nature of EST sequences [11,12]. However, the power of these resources is limited to analysis of the sequence features actually submitted to the databases [13]. For instance, EST sequences deposited in GenBank dbEST are typically trimmed of vector segments, adapter/linker sequences, insert-flanking restriction endonuclease recognition site, polyA and polyT tails prior to submission. Consequently, the above-mentioned terminus information is not available in the public EST databases. On the other hand, current sequence processing packages are somewhat limited in their capability of detecting and trimming such sequences with complete fidelity [14]. Moreover, these packages are not designed to use these trimmed sequence features in further analyses. Thus, the trimmed dbEST sequences present some obstacles for data quality control and validation of error-prone EST sequences, as well as data mining of sequence features with potential biological meanings whose detection relies on the terminus information in ESTs.

Both the NCBI Trace Archive [15] and Ensembl Trace Server [16] were established as public repositories for raw DNA sequencer traces that can be used to alleviate data mining limitations posed by trimmed sequences. As raw traces are increasingly being deposited in these repositories, there is a genuine need to reprocess trace files to detect both previously ignored and newly recognized sequence features with potential biological meaning. Unfortunately, these raw DNA traces have for the most part remained an untapped resource for general biologists due to the lack of freely available and easy-to-use bioinformatics tools for reprocessing raw traces, unambiguously identifying sequence features, and annotating 3' and/or 5' termini of cDNA inserts.

Using our novel, in-house software, WebTraceMiner [17], we have reprocessed 172,229 loblolly pine EST trace files downloaded from NCBI Trace Archive [15] and characterized and verified 3' and/or 5' termini of cDNA inserts *in silico*. Different from the most of other sequence processing packages, WebTraceMiner first detects all vector fragments, restriction endonuclease recognition sites, adapter/linker sequences, and polyA and polyT runs as putative features in an unbiased fashion. In each sequence read, the putative features can be identified in single or multiple occurrences, as independent or concatenated, and with perfect or imperfect (*i.e*., mismatch, insertion or deletion) matching patterns. Based on the expected structures of directional cDNA library construction, WebTraceMiner then examines the location, order, distance, fidelity and orientation of the putative features and identifies *in-silico *verified features that characterize termini of cDNA inserts (*i.e*., 5TSS, 3TSS, 5TNS and 3TNS, see Figure 1A, 1B). Different from all other existing public EST resources, ConiferEST [18] provides biologists with unique, easy-to-use, web-based data filtration, visualization and mining tools to explore both putative and verified sequence features. These features provide valuable information for data quality control and validation of error-prone EST sequences, help identify data abnormalities including EST chimeras, and facilitate detection of new potential functional motifs embedded in large EST datasets. Furthermore, sequence reads with verified features are also scanned for protein domain signatures using InterProScan [19] and the resultant data are made available for online exploration. Seamlessly integrated with other public EST resources, such as NCBI dbEST [11], Trace View [15], ORF Finder [20], UniGene [21] and Gene Indices [22], ConiferEST provides the community an invaluable and complementary resource for data validation, visualization and mining of previously ignored sequence features in growing datasets of conifer ESTs.

## Construction and Content

ConiferEST is composed of two major components: a relational database created using open-source MySQL 5.0 and a PHP web application that communicates with the database. All data in the database were primarily created from reprocessing raw DNA trace files using our in-house, freely available software, WebTraceMiner [17].

### Database

The ConiferEST database was designed for simplicity, efficiency and scalability. The database design has been carried out using Unified Modelling Language (UML) [23]. The core class is **SeqRead**, which describes the sequence reads obtained from processing raw sequencer trace files using Phred [24] or some other base caller. The trace files are characterized by **Trace**. Each sequence read is uniquely associated with one particular configuration of trace processing, represented as the class, **Config**. **Config **contains detailed information about how the trace files were processed (*e.g*., programs, program versions and relevant parameters used). All putative sequence features, including vector segments, restriction endonuclease recognition sites, adapter sequences, polyA/polyT runs and their locations (*i.e*., start and stop positions), fidelities (*i.e*., perfect or imperfect/fuzzy match patterns), and orientations (*e.g*., direct, palindromic or reverse-complemented matches), are characterized by the class, **FeaturePutative**. In contrast, the verified sequence features, such as 5' termini in the non-sense strand (*i.e*., authenticated 3' polyT tails), are defined in **FeatureVerified**. Every trace file is uniquely associated with a particular cDNA library represented as **Library**, which belongs to a specific species. The class, **Species**, has a one-to-many relationship with **Library**. For data integration, we also created classes for representing Gene Index [22] and InterProScan [19] annotation. As the system expands, more classes will be added to the database design.

### Data Integration

Currently, ConiferEST integrates information from NCBI dbEST [11], Trace View [15], ORF Finder [20] and Pine UniGene [21], as well as the Pine Gene Index [22] (see Figure 2A). The integration is mainly accomplished through data localization and creation of computer programs that can dynamically access relevant websites through their APIs (Application Programming Interfaces). For instance, we have incorporated NCBI ORF Finder dynamically using a PHP program. Upon a user's request, the cleaned portion of a given sequence read is automatically sent to the NCBI ORF Finder web site for 6-frame ORF detection. The user can then explore the ORF details using the relevant graphic web interface (see Figure 2A, 2D).

In particular, we have developed a high-throughput Perl program that identifies the best ORF for each sequence read having the expected *in-silico *verified sequence features. The filtration criteria include: (1) for all ESTs whose ORF start position is greater than 3, the ORFs must start with a start codon, and this requirement is waived for all ESTs whose ORF start position is less than or equal to 3; (2) for all ESTs, the ORF stop position should end with a stop codon; and (3) if there are multiple ORFs in a given sequence read that pass the first two criteria, the program will automatically pick the one with the maximum length. Subsequently, each ORF is scanned by InterProScan (version 4.2) to obtain protein domain signatures [19,25]. In order to gather more information, we included all InterPro member databases – UniProt, PROSITE, Pfam, PRINTS, ProDom, SMART, TIGRFAMS, PIRSF, SUPERFAMILY, Gene3D and PANTHER for scanning. We adopted the scanning methods of InterPro [26] using InterPro database release 13.1 plus PANTHER release 12.1. For 43,857 peptides identified in the current ConiferEST release, 27,104 retreived InterProScan annotation results, 19,218 retrieved InterPro entries, 17,290 retreived Pfam domains, and 14,001 retreived GO term annotations [27]. ConiferEST is constantly integrating additional data and tools to provide a dynamic community resource for conifer genomics.

## Utility and Discussion

As of March 2007, there were 42,050,137 entries deposited in the GenBank dbEST [11]. While these data are being widely utilized for characterizing the active, protein-coding portions of various genomes, they also present a serious challenge for data quality control and validation due to the inherent deficiencies of EST sequences. On the other hand, as genomic research deepens, there is an increasing need for the capacity to reprocess EST traces in order to inspect sequence features that have previously been ignored (*e.g*., 3' and/or 5' termini) and detect new features that have potential biological meaning. For instance, mRNA polyA length proves to be related with its stability [28]. Recently, polyA tails in 5'-transcript ends have been reported [29]. Without doubt, the 3' and 5' termini in ESTs and their associated information can be critical for such EST applications as 3'- and 5'-UTR determination, annotation of genes, and identification of chimeric ESTs. Unfortunately, substantial terminus information is not available in the current public EST resources. Consequently, ConiferEST was designed to be a unique and complementary EST resource that can fill these gaps by focusing on the previously ignored features for the purposes of data quality control, validation and integration and for the possibility to explore new potential functional motifs embedded in large EST datasets.

In its current release, Build 1.0, ConiferEST contains 172,229 loblolly pine raw EST traces downloaded from NCBI Trace Archive [15]. Sequence reads from these trace files were previously screened for vector sequence and other contamination, including *Escherichia coli*, mitochondria, and chloroplast genomic DNA, as well as *r*RNA, trimmed for polyT tails and adapter/linker sequences, and deposited in dbEST. Among those previously processed reads, 83,021 were categorized as 3' EST sequences, and 89,208 were designated 5' EST sequences. After reprocessing with WebTraceMiner, only 30.03% of the designated 3' EST sequences were found to have an authenticated 5' terminus in the non-sense strand (*i.e*., 5TNS, authenticated polyT tails consistent with the expected structures), while fewer than 5.34% of the designated 5' EST sequences had an *in-silico *verified 5' terminus in the sense strand (*i.e*., 5TSS)(see Table 1). This is consistent with the knowledge that current cDNA library construction protocols are biased against 5' UTRs. Also, as shown in the Table 1, only about 3.16% of the designated 5' EST sequences had authenticated 3' termini in the sense strand (*i.e*., 3TSS, authenticated polyA tail, consistent with the expected structures). These sequences mostly represented the products of short genes and mRNA transcripts truncated at their 5' ends.

Oligo-dT is commonly used to prime the reverse transcriptase reactions that generate cDNAs for EST library preparation. As a consequence, most, if not all, 3' EST sequences should, in theory, harbour polyT tails at their ends. However, in practice, such polyT tails are not always present. This can occur if the oligo-dT primer misprimes from a stretch of adenosyl residues internal to the mRNA transcript, but can also arise from DNA sequencing issues, such as sequencing primers that are positioned too closely to the end of cDNA inserts to yield accurate reads, or low-quality sequence yielded from single-pass sequencing. (Note also that total numbers of 3' ends containing polyT sequences are likely under-reported for many EST projects since overly long reads of polyT homopolymer are usually discarded as 'failed' reads.) Since most sequences submitted to dbEST are trimmed of polyT/A tails before submission, and information about the presence or absence of polyT/A tails is not mandatory for submission, it is a common assumption that all reported 3' ESTs had polyT tails. However, as shown in our analyses of the loblolly pine trace files, only about 30.03% of the 3' ESTs possessed authenticated polyT tails. As per the expected structures for the loblolly pine libraries, *in-silico *verified polyT tails are currently defined in ConiferEST as those immediately following an XhoI restriction site, either in perfect or imperfect matching patterns, with an allowance for a minimal number of low-quality nucleotide bases between the polyT tails and restriction sites. The ability to identify these verified polyT tails can have a fundamental impact on certain downstream EST data analyses, such as annotation of gene ends and detection of polyadenylation signals. Recent studies show that alternative polyadenylation is very important in post-transcriptional gene expression and regulation [30,31]. Unambiguous identification of polyA sites in ESTs can provide critical positional information for identification of relevant polyadenylation and/or alternative polyadenynation signals, which are usually located upstream and within a certain distance of the polyA sites in plant genes (in animals, there is an additional downstream signalling element). *In-silico *detection of polyadenylation sites may also prove useful for identifying non-templated nucleotide addition prior to polyadenylation [32], as well as base substitutions within the polyA tails that are not due to sequencing errors but, in fact, might have biological meaning. ConiferEST provides a unique tool for examining trace file datasets for such novel sequence features.

**Figure 3:**
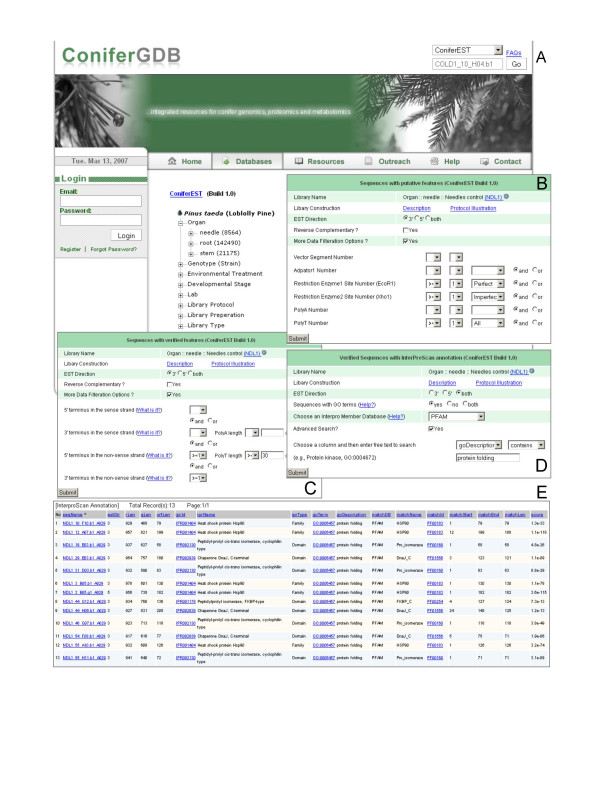
**Snapshots of ConiferEST Web Portals**. **Panel A**: All cDNA libraries of *Pinus taeda *have been differentially categorized for easy data navigation and comparison. For fast retrieval of individual sequences, users first select the ConiferEST option within the pull-down menu shown in the top portion. Users then enter either the specific sequence name (*e.g*., FLD1_34_H08.g1_A029), GenBank accession (*e.g*., CO162374), or GenBank gi number (*e.g*., 48932915), and click the *Go *button. **Panel B**: After choosing one or more libraries from the expandable tree shown in Panel A, the database query interface provides users three options, one of which is "Sequences with putative features". As shown, there is a variety of data filters that can be applied to retrieve putative feature data in terms of users' needs. **Panel C**: The second option is "Sequences with verified features". Users can not only specify sequences with or without certain verified termini, but also require specific length for verified polyA and/or polyT tails. **Panel D**: The third option is "Sequence with InterproScan annotation". Users can choose among different InterPro Member databases. They can also conduct advanced field search by text pattern matching. **Panel E**: Clicking the *Submit *button shown in the Panel D returns the sortable, tabulated InterProScan results from the database.

To the best of our knowledge, ConiferEST is the first public resource that allows biologists to explore EST data with respect to terminus structure, as well as related data abnormalities, with easy navigation, powerful search and sophisticated visualization. At the main entry portal for ConiferEST (Figure 3A), all cDNA libraries have been categorized with respect to a variety of parameters in order to enhance the efficiency of database searching and data comparison. Thus, the libraries have been categorized according to the laboratory that prepared the cDNA samples, the library protocol (*e.g*., subtracted/normalized library versus normal library), the library preparation (*e.g*., directional cloning) and library type (*e.g*., EST or not). In addition, the cDNA libraries have also been grouped according to organ, developmental stage, and genotype, three of the most important factors affecting gene regulation and expression. Users can select either a single cDNA library or a group of libraries within a specific category. Following the main entry portal, there are three major web portals available: one for "Sequences with putative features" (Figure 3B), one for "Sequences with verified features" (Figure 3C) and one for "Sequences with InterProScan Annotation" (Figure 3D). Using these three web portals, users can easily customize their data search or filtration methods to explore data that is interesting to them. For example, users can retrieve all sequences which have an authenticated polyT tail with a certain length threshold (*e.g*., > = 30) by customizing the settings shown in the "Sequences with verified features" web portal (Figure 3C). Similarly, the user can even find out how many 3' sequences have a verified polyA tail (*i.e*., 3TSS, 3' terminus in the sense strand) or how many 5' sequences have a verified polyT tail (*i.e*., 5TNS, 5' termini in the non-sense strand). The ability of ConiferEST to systematically present complex EST data to the community in a searchable browser format is a major strength of the system.

As common and problematic EST abnormalities, cDNA chimeras have been reported on numerous occasions [33,34]. Substantial work has been done to detect chimeric ESTs computationally; however, a majority of these systems rely on genomic sequence information [35-37]. Through preliminary data analysis, we found that "double-termini adapters" have great potential to identify chimeric ESTs, as well as other abnormalities. For instance, as shown in Figure 1D, COLD1_32_H06.b1_A029 is designated as a 3'-end sequence. However, it actually contains a 5'-like sequence as judged from its terminus structure. Specifically, four different termini (*i.e*., 5TSS, 3TSS, 5TNS and 3TNS) coexist in this sequence read where they form a "double-termini assembly". Also, as shown in Figure 2A, both the 3'-end COLD1_10_H04.b1_A029, displayed as a reverse complement view, and its 5'-end counter part sequence, COLD1_10_H04.g1_A029, contain the "double-termini adapters" structure to join two different transcripts and form a chimeric cDNA insert. Utilizing Gene Index cluster information (TC65773), a user can verify that these two cDNA sequences containing "double-termini adapters" cluster with many different cDNAs (Figure 2C). It is obvious that the cluster contains more than 300 additional sequences that match the false 3' end. About 3% of the EST sequence reads in this release contain such "double-termini adapters". After examining many examples, we concluded that "double-termini adapters" were a very good indicator of potential EST chimeras. Further work needs to be done in this area to see whether this is a universal phenomenon. Readers may recover the sequences with "double-termini adapters" for online exploration by setting both "5' terminus in the sense strand" and "3' terminus in the non-sense strand" greater than 1 in the "Sequence with verified features" web portal (Figure 3C).

Genomic information available online is increasing rapidly, but the information is often isolated and scattered among different websites and locations. There is a genuine need for biologists to have integrated resources so that they can easily obtain the most useful information for their research. By integrating data from many different resources, ConiferEST provides biologists with a portal where they can sweep data in from numerous sites. For example, when a user finishes data filtration in "Verified Sequence with IntroProScan Annotation" web portal, shown in Figure 3D, and clicks the "submit" button, a tabulated result page is displayed (Figure 3E). In the returned table, each column can be sorted independently to facilitate personalized searching. A Scalable Vector Graph (SVG) graph, as shown in Figure 1B, provides on-the-fly viewing for inspection of individual nucleotides and their quality after users click a sequence name shown in the table (Figure 3E). The SVG graphs and color-coded sequences can be redrawn with different zooming scales, and with or without space separators, to facilitate searching and text capture for other tools, such as BLAST [38]. In addition, for fast retrieval of individual sequences, users first select the ConiferEST option within the pull-down menu shown in the top portion of Figure 3A. Users then enter either the specific sequence name (*e.g*., FLD1_34_H08.g1_A029), GenBank accession (*e.g*., CO162374), or GenBank gi number (*e.g*., 48932915), and click the *Go *button. In both the *Putative *and *Verified Sequence Control Panel*, displayed at the top of Figures 2A, 2B, several additional data options are available for users to use in mining various data in both internal and external databases. Through the *Detailed Data *link, users can obtain individual Phred quality scores for each base, or reverse complement sequences by applying the relevant menu items, like *Sequence Quality *or *Reverse Complement*. Users can also toggle between views for all putative sequence features or for verified sequence features by clicking the menu items, *All Putative Features *or *Verified Features*. The *Sequence Feature Table *menu item inside *Detailed Data *brings users a detailed list of information about each sequence feature, either putative or verified, including start and stop positions, length, identity percentage (*i.e*., 100 stands for perfect matches) and match orientation (*i.e*., D indicates normal direction, P represents palindrome or reverse complement, and 0 is indeterminant). With the *Data Integration *link from within the *Putative*/*Verified Sequence Control Panel*, ConiferEST provides users a menu list that can result in direct access to a specific external resource for data integration (Figure 2A). Currently, this menu list contains items like *dbEST Accession*, *Trace View*, *UniGene*, *Gene Index*, *ORF *and *InterProScan*. In addition, several BLAST tools, including the BLAST ConiferEST, BLAST TAIR [39] and BLAST Populus [40], have been integrated through the *Other Tools *link within the *Putative/Verified Sequence Control Panel*. Additional tools for data mining will be added through the *Other Tools *link in the future.

## Conclusion

ConiferEST was designed to reprocess conifer traces to obtain better annotation of all raw sequence features, including 3' and 5' termini in both sense and non-sense strands, for the purpose of data quality control, validation and integration and for the possibility to explore new potential functional motifs embedded inside large EST datasets. Using the user-friendly web interfaces provided in ConiferEST, biologists can easily navigate, search, filter, visualize all putative and *in-silico *verified features, as well as InterProScan annotation for verified features. To the best of our knowledge, ConiferEST is the first public resource that reprocesses raw EST traces in large-scale by focusing on 3' and 5' termini of cDNA inserts and presents the EST sequences in a format that can be searched and visualized in a browser. It provides the biological community with a unique database tool that will complement existing public resources for mining of conifer EST sequences and affiliated information.

The categorization of sequence reads with respect to the expected cDNA insert structure reflecting cDNA library construction protocols is a complex procedure due to the numerous variations that can be made in the molecular biology steps employed for cDNA library construction. Efforts are underway to improve the ConiferEST classifier system so that it can categorize and finalize with better accuracy sequence reads with different termini combinations. In the near future, additional downstream analyses will be available in ConiferEST. For example, we are in the process of developing a ConiferEST GO Tree that will help users understand the ConiferEST GO [27] annotation in a more intuitive manner. In addition, we have started to work on EST re-clustering, with particular emphasis on the problematic EST chimeras. Continuous improvement of ConiferEST to integrate more data, functionality and search tools for biologists will make this database an invaluable resource for the plant genomic community.

## Authors' contributions

CL managed project development, designed the whole system, and drafted the manuscript. GW carried out web design and development and participated in database design, management and performance tuning. LL, KC and JSW worked on web development as well as testing and deploying. GJ and YL participated in improving the classifier system for authenticated sequence features. LF contributed to ORF detection and InterProScan. JFDD contributed to conceptualizing the biological system and participated in manuscript writing.

## Availability and requirements

The ConiferEST resource can be accessed via .

Requirement: The ConiferEST web interfaces work best with Firefox (, version 1.5 or above), a free web browser that provides better security and performance with bundled SVG (Scalable Vector Graphs) viewer plug-in. Our website also works with Internet Explorer (6.0 or above), but you need to download and install SVG viewer plug-in  by yourself.

Contact: Dr. Chun Liang at liangc@muohio.edu

## Figures and Tables

**Table 1:** Characterization of sequences with *in-silico *verified termini

		**Only One Terminus**^**2**^				**Only Two Termini**				**At Least One Terminus**			
		
	Num^1^	**5TSS**	**3TSS**	**5TNS**	**3TNS**	**5TSS+3TSS**	**5TNS+3TNS**	**5TSS+3TNS**	**5TNS+3TSS**	**5TSS**	**3TSS**	**5TNS**	**3TNS**
**3' reads**	83,021 (48.2%)	730 (0.88%)	80 (0.10%)	21,901 (26.38%)	6,574 (7.92%)	2 (0.00%)	2,306 (2.78%)	1,654 (1.99%)	7 (0.01%)	4,473 (5.39%)	161 (0.19%)	24,929 (30.03%)	12,642 (15.2%)
**5' reads**	89,208 (51.8%)	3,078 (3.45%)	2,632 (2.95%)	346 (0.39%)	614 (0.69%)	120 (0.13%)	3 (0.00%)	1,238 (1.39%)	14 (0.02%)	4,760 (5.34%)	2,815 (3.16%)	393 (0.44%)	2,172 (2.43%)

**Total**	172,229 (100%)	3,808 (2.21%)	2,712 (1.57%)	22,247 (12.92%)	7,188 (4.17%)	122 (0.07%)	2,309 (1.34%)	2,892 (1.68%)	21 (0.01%)	9,233 (5.36%)	2,976 (1.73%)	25,322 (14.70%)	14,814 (8.60%)

1. Num stands for the number of sequence reads and percentage (in parentheses) of either the 3'-, or 5'-reads to the total number of reads.

2. 5TSS = 5' terminus in the sense strand, 3TSS = 3' terminus in the sense strand, 5TNS = 5' terminus in the non-sense strand and 3TNS = 3' terminus in the non-sense strand.

All these data are retrievable through ConiferEST web interfaces (see Figure 3C, for example).
